# Fractures in CKD predialysis: the role of bone biopsy, biomarkers, and FRAX^®^

**DOI:** 10.1590/21758239-JBN-2025-E016en

**Published:** 2025-12-01

**Authors:** Aluízio Barbosa Carvalho

**Affiliations:** 1 Universidade Federal de São Paulo, São Paulo, SP, Brazil.

Bone fragility is a central concern in chronic kidney disease (CKD), contributing substantially to morbidity, mortality, and healthcare costs. The interplay between renal osteodystrophy (ROD), mineral metabolism disorders, and skeletal fragility complicates the clinical evaluation of fracture risk in this population^1^. In this issue of the *Brazilian Journal of Nephrology*, Diz-Lopes et al.^2^ present a timely study addressing the contribution of bone histomorphometry, circulating biomarkers of bone turnover, and the FRAX® tool in predicting fractures in predialysis CKD.

## Key Findings

Over a median follow-up of 7.5 years, the authors observed that nearly 10% of patients experienced a fracture, corresponding to an incidence of 12/1000 patient-years—higher than that reported in the general Portuguese population^3^. This finding underscores the recognized vulnerability of patients with CKD to skeletal fragility, even before the initiation of dialysis.

Interestingly, histomorphometric subtypes of ROD were not associated with fracture risk, nor were circulating bone biomarkers, such as PTH, FGF- 23, sclerostin, DKK-1, RANKL, or osteoprotegerin. These results challenge the assumption that categorizing bone turnover and mineralization disturbances is sufficient to stratify skeletal risk. Instead, the data suggest that the microstructural determinants of bone integrity may be more relevant than static histological subtypes. Supporting this interpretation, there was a tendency toward lower bone volume in patients with fractures, highlighting that quantitative structural deficits may be more clinically meaningful than categorical histology alone^4^.

In contrast, the FRAX® tool, even when calculated without bone mineral density (BMD), showed good discriminative performance for fracture risk. Significantly, its sensitivity improved when CKD was included as a cause of secondary osteoporosis. This is a clinically relevant observation: DXA, the standard method for osteoporosis evaluation in the general population, has recognized limitations in CKD due to vascular calcification, altered bone quality, and other confounding factors^5^. A bone biopsy, while providing unparalleled information on bone turnover, mineralization, and volume, is an invasive procedure and is seldom performed outside of research centers^4^. FRAX^®^, therefore, emerges as a pragmatic and accessible instrument, potentially bridging the gap between insufficient tools and the clinical need for reliable fracture risk prediction^6^.

## Clinical Implications

The results highlight two critical aspects of fracture risk assessment in CKD:

1. **Histology and biomarkers alone are insufficient.**While bone biopsy remains the gold standard for assessing renal osteodystrophy, its findings in this cohort did not predict fractures. Likewise, circulating biomarkers failed to discriminate risk. This reinforces that skeletal fragility in CKD is a multifactorial phenomenon, not entirely captured by turnover or mineralization status^4,5^.
2. **FRAX** ^®^ **is a pragmatic and helpful tool.**The ability of FRAX^®^ to identify patients at higher risk—even in the absence of BMD data—offers clinicians a practical alternative for daily practice. Adjusting FRAX^®^ by accounting for CKD as secondary cause of osteoporosis enhanced its sensitivity, which is particularly valuable in a population where fracture risk is often underestimated^6^.

## Limitations and Perspectives

This study is not without limitations. Its retrospective design and modest sample size reduce statistical power and generalizability. The heterogeneity of the cohort, with some patients progressing to dialysis while others remained in stages 3–4 CKD, introduces variability that may influence fracture outcomes. Moreover, bone biopsies and biomarkers were assessed only at baseline, whereas fracture risk is dynamic and evolves with the decline in renal function. These constraints highlight the challenges of conducting fracture risk research in CKD, where invasive procedures, extended follow-up periods, and heterogeneous populations often limit conclusions.

Nonetheless, this work is among the few to directly explore the interplay between ROD subtypes, bone biomarkers, and FRAX^®^ in predialysis CKD, and it adds valuable evidence to a field where data remain scarce. Importantly, it suggests that fracture prediction may rely less on static measures of turnover or single biomarkers, and more on integrative tools that combine clinical risk factors with population- derived fracture probabilities.

Future research should prioritize prospective, multicenter studies with larger cohorts, ideally integrating longitudinal bone biopsies, serial biomarker assessments, and advanced imaging modalities. Techniques such as high-resolution peripheral quantitative computed tomography (HR-pQCT) may provide novel insights into bone microarchitecture and quality beyond BMD and histology^7^. Comparative studies between FRAX^®^, DXA, and histomorphometry could clarify their relative contributions and guide a stepwise approach to fracture risk assessment in CKD.

## Conclusion

Diz-Lopes et al.^2^ make a meaningful contribution to the ongoing discussion on how to best assess skeletal health in CKD. Their findings suggest that fracture risk cannot be fully explained by bone histology or biomarkers alone, and that FRAX® offers a valuable, clinically applicable tool in predialysis CKD, particularly when adjusted for secondary osteoporosis.

As nephrologists increasingly recognize the burden of fragility fractures in CKD, the integration of FRAX^®^ into routine care may help bridge the gap between the limited accessibility of bone biopsy and the shortcomings of DXA. Ultimately, skeletal evaluation should become as integral to CKD management as cardiovascular and metabolic care. Preventing fractures must be regarded not as an ancillary goal but as a central component of comprehensive renal care.

## Data Availability

No new data was generated or analyzed in this study.
