# Supplementary material for: Fractures in CKD predialysis: the role of bone biopsy, biomarkers, and FRAX®
Source: J Bras Nefrol. 2025 Dec 1;47(4):e2025E016. doi: 10.1590/21758239-JBN-2025-E016en (PMC12668541; doi:10.1590/21758239-JBN-2025-E016en)
Supplement: Translation

# Translation: Fraturas na DRC pré-diálise: o papel da biópsia óssea, biomarcadores e FRAX^®^

**DOI:** 10.1590/21758239-JBN-2025-E016pt

**Authors:** Aluízio Barbosa Carvalho

**Affiliations:** 1 Universidade Federal de São Paulo, São Paulo, SP, Brasil.

A fragilidade óssea é uma questão central na doença renal crônica (DRC), contribuindo substancialmente para a morbidade, mortalidade e custos em saúde. A interação entre a osteodistrofia renal (ODR), os distúrbios do metabolismo mineral e a fragilidade esquelética complica a avaliação clínica do risco de fraturas nessa população^1^. Nesta edição do *Jornal Brasileiro de Nefrologia*, Diz-Lopes et al.^2^ apresentam um estudo oportuno que aborda a contribuição da histomorfometria óssea, dos biomarcadores séricos de remodelação óssea e da ferramenta FRAX® na predição de fraturas em pacientes com DRC pré-diálise.

## Principais Achados

Com um seguimento médio de 7,5 anos, os autores observaram que cerca de 10% dos pacientes apresentaram fraturas, o que corresponde a uma incidência de 12/1000 pacientes-ano — valor superior ao relatado na população portuguesa geral^3^. Esse achado reforça a reconhecida vulnerabilidade dos pacientes com DRC à fragilidade esquelética, mesmo antes do início da diálise.

Interessantemente, os subtipos histomorfométricos de ODR não se associaram ao risco de fraturas, tampouco os biomarcadores ósseos séricos, como PTH, FGF-23, esclerostina, DKK-1, RANKL ou osteoprotegerina. Esses resultados desafiam a suposição de que a categorização das alterações de remodelação e de mineralização ósseas seja suficiente para estratificar o risco esquelético. Em vez disso, os dados sugerem que os determinantes microestruturais da integridade óssea possam ser mais relevantes do que os subtipos histológicos estáticos. Sustentando essa interpretação, observou-se uma tendência a menor volume ósseo nos pacientes com fraturas, indicando que déficits estruturais quantitativos podem ter maior significado clínico do que a categorização dos tipos histológicos isoladamente^4^.

Em contraste, a ferramenta FRAX®, mesmo quando calculada sem a densidade mineral óssea (DMO), apresentou bom desempenho discriminatório para risco de fraturas. De forma relevante, sua sensibilidade aumentou quando a DRC foi incluída como causa de osteoporose secundária. Essa é uma observação de grande relevância clínica: a DMO por DXA, método padrão para avaliação de osteoporose na população geral, apresenta limitações reconhecidas na DRC devido à calcificação vascular, à qualidade óssea alterada e a outros fatores confundidores^5^. A biópsia óssea, embora forneça informações incomparáveis sobre remodelação, mineralização e volume ósseo, é um procedimento invasivo e raramente realizado fora de centros de pesquisa^4^. Assim, o FRAX® desponta como um instrumento pragmático e acessível, capaz de preencher a lacuna entre ferramentas insuficientes e a necessidade clínica de uma predição confiável do risco de fraturas^6^.

## Implicações Clínicas

Os resultados destacam dois aspectos críticos na avaliação do risco de fraturas na DRC:

1. **Histologia e biomarcadores, isoladamente, são insuficientes.**Embora a biópsia óssea permaneça o padrão-ouro para a avaliação da ODR, seus achados nesta coorte não foram capazes de prever fraturas. Da mesma forma, os biomarcadores ósseos séricos não conseguiram discriminar o risco. Esses resultados reforçam a noção de que a fragilidade esquelética na DRC é um fenômeno multifatorial, não totalmente captado pelo estado de remodelação ou mineralização^4,5^.
2. **O FRAX® é uma ferramenta prática e útil.**Sua capacidade de identificar pacientes com maior risco — mesmo na ausência de dados de DMO — oferece aos clínicos uma alternativa aplicável à prática diária. O ajuste do FRAX® considerando a DRC como causa de osteoporose secundária aumentou sua sensibilidade, o que é particularmente valioso em uma população cujo risco de fraturas é frequentemente subestimado^6^.

## Limitações e Perspectivas

Este estudo não está isento de limitações. Seu desenho retrospectivo e o tamanho amostral modesto reduzem o poder estatístico e a generalização dos resultados. A heterogeneidade da coorte, com alguns pacientes progredindo para diálise enquanto outros permanecem nos estágios 3–4 da DRC, introduz variáveis que podem influenciar os desfechos de fratura. Além disso, as biópsias ósseas e os biomarcadores foram avaliados apenas no início do estudo, embora o risco de fraturas seja dinâmico e evolua conforme o declínio da função renal. Essas limitações ressaltam os desafios inerentes à pesquisa sobre risco de fraturas na DRC, na qual procedimentos invasivos, longos períodos de seguimento e populações heterogêneas frequentemente limitam as conclusões.

Ainda assim, este trabalho está entre os poucos que exploram diretamente a interação entre os subtipos de ODR, biomarcadores ósseos e FRAX® na DRC pré-diálise, acrescentando evidências valiosas a um campo em que os dados permanecem escassos. De forma relevante, o estudo sugere que a predição de fraturas pode depender menos de medidas estáticas de remodelação ou de biomarcadores isolados e mais de ferramentas integradas que combinem fatores clínicos de risco com as probabilidades de fraturas nessa população.

Pesquisas futuras devem priorizar estudos prospectivos e multicêntricos, com coortes maiores, idealmente integrando biópsias ósseas seriadas, avaliações longitudinais de biomarcadores e modalidades avançadas de imagem. Técnicas como a tomografia computadorizada quantitativa periférica de alta resolução (HR-pQCT) podem oferecer novos *insights* sobre a microarquitetura e a qualidade óssea, somando-se às informações fornecidas pela DMO e pela histologia^7^. Estudos comparativos entre FRAX®, DXA e histomorfometria poderão esclarecer suas contribuições relativas e orientar uma abordagem escalonada para a avaliação do risco de fraturas na DRC.

## Conclusão

Diz-Lopes et al.^2^ oferecem uma contribuição significativa à discussão sobre a melhor forma de avaliar a saúde esquelética na DRC. Seus achados indicam que o risco de fraturas não pode ser plenamente explicado apenas pela histologia óssea ou pelos biomarcadores, e que o FRAX® representa uma ferramenta valiosa e clinicamente aplicável na DRC pré-diálise, especialmente quando ajustado para osteoporose secundária.

À medida que os nefrologistas reconhecem, cada vez mais, o impacto das fraturas por fragilidade na DRC, a incorporação do FRAX® à prática clínica pode ajudar a reduzir a lacuna entre a limitada acessibilidade da biópsia óssea e as deficiências da DXA. Em última análise, a avaliação esquelética deve tornar-se parte integrante do manejo da DRC, assim como o cuidado cardiovascular e metabólico. Prevenir fraturas deve ser considerado não como um objetivo acessório, mas sim como um componente central de um cuidado renal abrangente.

## Data Availability

Nenhum dado novo foi gerado ou analisado neste estudo.
